# Kinship and similarity drive coordination of breeding-group choice in male spotted hyenas

**DOI:** 10.1098/rsbl.2022.0402

**Published:** 2022-12-14

**Authors:** Eve Davidian, Oliver P. Höner

**Affiliations:** ^1^ Ngorongoro Hyena Project, Ngorongoro Conservation Area, Arusha, Tanzania; ^2^ Department of Evolutionary Ecology, Leibniz Institute for Zoo and Wildlife Research, Berlin 10315, Germany

**Keywords:** behavioural synchronization, kin selection, sibling resemblance, parallel and collective dispersal, density dependence, spotted hyena

## Abstract

When and where animals reproduce influences the social, demographic and genetic properties of the groups and populations they live in. We examined the extent to which male spotted hyenas (*Crocuta crocuta*) coordinate their breeding-group choice. We tested whether their propensity to settle in the same group is shaped by passive processes driven by similarities in their socio-ecological background and genotype or by an adaptive process driven by kin selection. We compared the choices of 148 pairs of same-cohort males that varied in similarity and kinship. We found strong support for both processes. Coordination was highest (70% of pairs) for littermates, who share most cumulative similarity, lower (36%) among peers born in the same group to different mothers, and lowest (7%) among strangers originating from different groups and mothers. Consistent with the kin selection hypothesis, the propensity to choose the same group was density dependent for full siblings and close kin, but not distant kin. Coordination increased as the number of breeding females and male competitors in social groups increased, i.e. when costs of kin competition over mates decreased and benefits of kin cooperation increased. Our results contrast with the traditional view that breeding-group choice and dispersal are predominantly solitary processes.

## 1. Introduction

When and where individuals breed strongly influences their fitness and has far-reaching implications for social evolution [1,2] and the connectivity and resilience of populations [3,4]. Individuals often differ in their breeding decisions because these decisions are shaped by multi-level feedbacks and interactions between individual characteristics (i.e. genotype and phenotype), group- and population-level parameters (i.e. social organization, kin and demographic composition [5]), and the species' mating system [4,6–8]. Many theoretical and empirical studies have emphasized the causes of inter-individual heterogeneity in the propensity to disperse [6,9,10], the timing and quality of settlement decisions [11–13], and the trajectory or distance travelled [14–16]. By contrast, few studies examined what drives conspecifics to coordinate their breeding-group choices in space and time [17].

Coordinated dispersal and breeding-group choice, whereby subgroups of individuals jointly emigrate from their birth site and/or settle in the same or neighbouring breeding sites, have been reported in eusocial invertebrates, marine organisms and cooperatively breeding birds and mammals [17–26]. In these systems, dispersing groups usually consist of closely related individuals and coordination has been suggested to be shaped by kin selection. Yet, studies often lack the necessary control individuals and variance in demographic parameters and kinship to disentangle whether such coordination is driven by (i) adaptive processes of kin selection or (ii) passive, self-organized processes [27] shaped by similarities in preference or cognitive and physical capacities (similarity hypothesis) that may arise from a common socio-ecological background and genotype [17,18,24,28,29].

Here, we present the first account of coordination of breeding-group choice among male spotted hyenas *Crocuta crocuta*, a non-cooperatively breeding social carnivore that lives in multi-male–multi-female groups (clans) of up to 130 individuals [30,31]. We used 24 years of behavioural, demographic and genetic pedigree data from eight clans of a free-ranging population in Tanzania that is characterized by high local recruitment [10]. We tested the two hypotheses by comparing the settlement choices of pairs of males that belonged to the same cohort but varied in their degree of similarity in maternal upbringing, ecological environment and genotype, and in their kinship ties. ‘Littermates’—i.e. mostly full siblings, raised by the same mother in the same clan—shared highest cumulative similarity and strongest kinship ties; ‘peers’—i.e. close or distant kin of the same clan but different mothers—were less closely related and experienced a different maternal environment but a similar ecological environment; ‘strangers’—i.e. mostly distant kin, from different clans and mothers—were least related and similar.

According to the similarity hypothesis, littermates should show the highest propensity to choose the same breeding clan and to synchronize their choice [4,17], followed by peers and strangers. Also, the propensity of males to coordinate their choice should not be influenced by changes in the size of clans because similarities among pairs remain unchanged. By contrast, under kin selection, the coordination among full siblings and close kin should increase with increasing clan sizes because the trade-offs between kin competition and kin cooperation are density dependent [32,33]. Specifically, when the number of breeding females and male competitors is low, males benefit from avoiding kin competition by immigrating to different clans. As the number of breeding females and male competitors in clans increase, kin-mates benefit from coordinating their breeding-clan choice with a potential ally [32,33]. Coordination among distant kin should remain stable because changes in kin competition and kin cooperation and the resulting fitness benefits to coordinate breeding-clan choice are minimal.

## 2. Material and methods

### (a) Study population

Data were collected between April 1996 and December 2020 as part of the near-daily monitoring of the behaviour and life history of all individually known spotted hyenas of the eight clans inhabiting the Ngorongoro Crater in Tanzania [34,35]. Male and female clan members reproduce promiscuously [10,36]. Females give birth to litters of one or two (rarely three) cubs [37]; most litters—overall 84% [38]—are sired by one father. The same-cohort cubs born in the same clan are raised in a communal den and therefore are familiar with each other. Mother–offspring affiliation was assessed via observations of suckling interactions and confirmed by genetic analyses [39,40]; birthdates of cubs were estimated with an accuracy of ± 7 days [41]. Relatedness and kinship were derived from genetic pedigree information across nine generations. Over 80% of males born in the population choose one of the eight study clans to breed [10,39] and do so when 3.4 years old on average [10,13]; 85% of these males disperse from their birth clan and join another Crater clan as immigrant males while 15% establish themselves as reproducing ‘philopatric’ males in their birth clan [10]. Female philopatry is the norm [31,34]. Males were considered to have chosen a breeding clan when they displayed a sexual interest in the females and engaged in social interactions (see electronic supplementary material, S1) with the members of that clan for at least three months [35,42]. The date of clan choice was the date of first observation of such behaviour [10].

### (b) Pairing of males

We paired 296 males that chose a breeding clan during the study period. To control for the potential influence of age and the characteristics of dispersal destinations on clan choice [10,39] pairs consisted of males of the same cohort that were born within 60 days of one another. We assigned these pairs to one of three types: ‘littermates’ (*n* = 30 pairs) were brothers of the same litter; ‘peers’ (*n* = 63 pairs) were born within 17.7 ± 15.6 days in the same clan to different mothers; ‘strangers’ (*n* = 55 pairs) were born within 18.7 ± 15.0 days in different clans to different mothers. Males were paired once; pairs were treated as independent data (see electronic supplementary material S1).

To disentangle the effects of similarity in socio-ecological background and genotype, pairs were further categorized according to male origin (same or different birth clan) and degree of kinship, as assessed from their coefficient of relatedness (*r*) calculated along their maternal and paternal lineages [43]. We considered three categories of kin: (i) full siblings (*r* = 0.5, *n* = 27 pairs), (ii) close kin (0.5 > *r* ≥ 0.125, *n* = 29) or (iii) distant kin (*r* < 0.125, *n* = 92).

### (c) Maternal upbringing

The maternal rank in the clan dominance hierarchy defines the quality of the postnatal environment [37] and shapes the life history and fitness of sons, including the quality of their breeding-clan choice, as defined by the number of breeding females in the clan upon clan choice [10,13,39]. To account for this effect, we calculated within-pair differences in maternal ranks; these ranks range from −1 (lowest rank) to +1 (highest rank) (for details see [35]). Littermates were assigned a rank difference of 0; the absolute difference in maternal rank was 0.55 ± 0.37 (range = 0–1.35) among peers and 0.45 ± 0.36 (range = 0–1.60) among strangers.

### (d) Mean clan size

We used mean clan size as an integrative proxy for male–male competition over social integration into a clan and access to mates. For each male pair, we calculated the mean clan size as the total number of adult females (i.e. breeding partners) plus philopatric and immigrant males (i.e. competitors) in the population, averaged over the eight study clans, on the date of clan choice by the member of the pair who expressed his choice last. This proxy is adequate because sex ratio in clans is balanced and clan sizes are positively correlated (*R*^2^ = 0.62; see electronic supplementary material, figure S1).

### (e) Statistical analyses

Data extraction [44] and statistical analyses were conducted using R statistical software v. 4.2.0 [45]. Tests were two-tailed (where applicable) and the threshold for significance was alpha = 0.05. Synchronization of breeding-clan choice of littermates, peers and strangers was compared using non-parametric tests. Factors influencing the probability of paired males to choose the same breeding clan were modelled using logistic regression with a binomial logit link using function *glm()* [46]. Origin, difference in maternal rank, kinship, mean clan size and their interaction were included as predictors. Mean clan size was scaled prior to modelling to satisfy model assumptions. Model assumptions were verified using function *simulateResiduals()* [47]; absence of multi-collinearity was confirmed by squared generalized variance inflation factors (GVIF^[1/(2*df)])^2 [48,49]. Model estimates were back-transformed as odds ratios and plotted using function *plot_model()* [50].

## 3. Results

**Figure 1.**
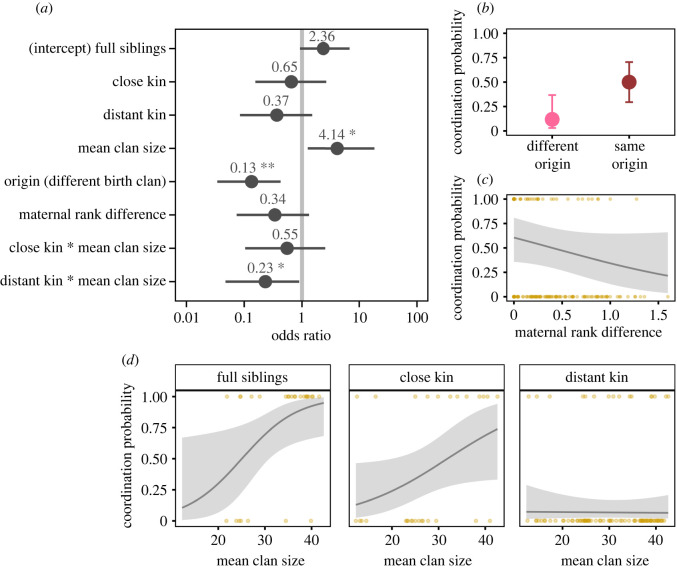
Factors influencing the propensity of paired male spotted hyenas to choose the same breeding clan. (*a*) Model estimates as odds ratios ±CI_95%_ for each covariate as derived from a logistic regression. (*b–d*) Predicted coordination probabilities ±CI_95%_ for males originating from the same or different clans, males of varying similarity in maternal upbringing and males of three categories of kinship as a function of mean clan size. Predictions are for close kin, mean clan size and difference in maternal rank at population average (*b*), for close kin, same origin and mean clan size at population average (*c*), and accounted for biological differences between kin categories: full siblings had same origin and rank difference = 0, close kin had same origin and rank difference = 0.5, distant kin had different origin and rank difference = 0.45 (*d*). Asterisks indicate levels of significance for estimates; *: *p* < 0.05, **: *p* < 0.01.

Overall, littermates showed the highest spatial coordination, with 70% (21 out of 30 pairs) choosing the same breeding clan, followed by 36.5% of peers (24 out of 63 pairs) and 7.3% of strangers (4 out of 55 pairs). Male origin strongly affected the propensity of male pairs to choose the same clan (figure 1*a*; see electronic supplementary material, table S1 for detailed model results); males born in the same clan were more likely to coordinate their choice than males born in different clans, when controlling for covariates (figure 1*b*). The propensity of males to choose the same clan also decreased with increasing difference in their maternal rank, although the effect was not statistically significant (figure 1*a*,*c*).

Mean clan size affected male coordination differently across the three kinship categories (likelihood ratio: 12.9, *p* = 0.024; figure 1*a*). Full siblings (slope coefficient = 1.42, CI_95%_ = 0.13 to 2.71; figure 1*d*) and to a lesser extent close kin (slope = 0.83, CI_95%_ = −0.03 to 1.68) were more likely to choose the same clan as mean clan size increased. By contrast, breeding-clan choices of distant kin were unaffected by demographic changes (slope = −0.04, CI_95%_ = −0.65 to 0.57).

**Figure 2.**
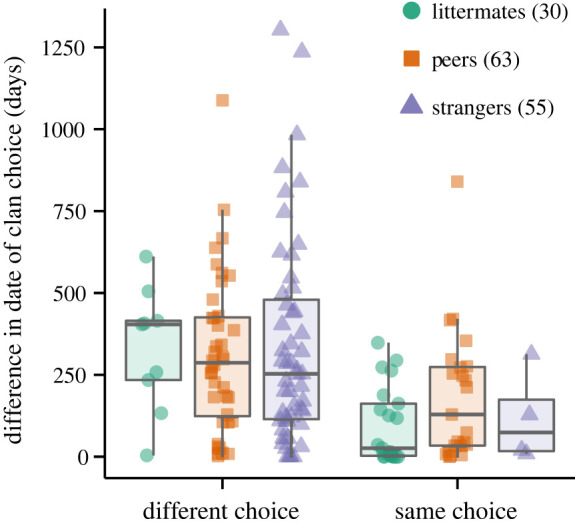
Temporal coordination of paired male spotted hyenas depending on whether they chose the same or different breeding clans. Boxes indicate the interquartile range around the median (horizontal line), vertical bars represent timing differences that lie within 1.5 times the interquartile range of the raw data (filled symbols). Numbers in brackets indicate the number of pairs.

The three types of male pairs differed in the temporal coordination (or ‘synchronization’) of their breeding-clan choices (Kruskal–Wallis rank sum test; *χ*^2^ = 7.2602, d.f. = 2, *p* = 0.027), with littermates showing higher synchronization than strangers (Dunn *post hoc* test, *p* = 0.002). Synchronization of littermates and peers and of peers and strangers was similar (*p* > 0.05). Synchronization also varied with the spatial coordination of breeding-clan choices (Mann–Whitney *U*-test; *U* = 3510.5, *p* < 0.0001; figure 2); littermates were more synchronized when they chose the same clan (within-pair difference in date of clan choice = 96.34 ± 116.36 days, *n* = 21 pairs) than when they chose different clans (330.11 ± 189.64 days, *n* = 9 pairs; *U* = 159.5, *p* = 0.003).

## 4. Discussion

Our results provide the first demonstration of coordination of breeding-clan choice by male spotted hyenas. Coordination was particularly striking among littermates but also apparent among peers, that is, close or distant kin of the same birth clan. The coordination of male breeding-clan choice likely emerged from a combination of passive processes driven by similarities in needs, capacities and breeding options, and an adaptive and flexible decision process shaped by kin selection.

Consistent with the similarity hypothesis, male coordination was influenced by similarities in genotype, origin and maternal environment. Furthermore, the propensity of two males to choose the same breeding clan increased with their cumulative similarity, with littermates showing the highest coordination, followed by peers and strangers. The strong influence of shared origin found here corroborates previous findings that breeding-clan choice in spotted hyenas is birthplace-dependent and shaped by the socio-ecological environment experienced while growing up and upon clan choice [10].

Consistent with the kin selection hypothesis, the coordination among full siblings and among close kin showed strong density dependence while that among distant kin did not. Full siblings and close kin were more likely to choose the same breeding clan as the number of breeding females and male competitors increased. This suggests that males actively adjust their settlement decisions to the dynamic trade-offs between kin competition and kin cooperation. Interestingly, full and half-siblings (i.e. littermates) who chose the same clan were highly synchronized, suggesting that they do not actively minimize tenure overlap and the potential reproductive cost of competing over the same pool of breeding females.

We cannot fully discard the possibility that the density dependence of the coordination among full siblings and close kin does not, at least in part, result from passive processes shaped by density-dependent genetic effects [51,52]. Yet, kinship is a strong driver of coalitionary support—a potent form of cooperation—in spotted hyenas [31,53]. The frequent coordination of breeding-clan choices therefore does create the potential for the evolution of cooperation among males [54,55]. Furthermore, cooperation may also operate between distantly related individuals that are familiar to one another, as in Assamese macaques, red squirrels, and Seychelles warblers [21,56,57]. Given that common clan membership and familiarity promote social bonds and alliances in spotted hyena society [31,53,58,59], peers may constitute an abundant source of potential allies. Settling with—or joining a clan that already contains—social allies may help overcome social resistance from already established males [60,61] and allow hyena males to settle in high-quality clans [13]. Allies may further function as a social buffer and help catalyse social integration and maintain social rank [31,35,59], which in hyena society renders high fitness benefits [10]. The extent and consequences of cooperation among male littermates and peers upon clan choice and afterwards remain to be investigated.

By showing that dispersal and breeding-group choice may often be coordinated, our findings contribute to the growing evidence [2,17,62] that coordinated breeding-group choice and dispersal is an overlooked driver in social evolution. Our study underscores the importance to account for kinship and similarity, in particular in origin, between dispersers in theoretical and empirical studies of animal movement and population genetics (see also [63]).

## Data Availability

Data are available from Figshare https://doi.org/10.6084/m9.figshare.21324393 [64]. Additional details about data collection and methods are available in the electronic supplementary material [65].
